# Recurrent Epileptic Auras As a Presenting Symptom of Alzheimer’s Disease

**DOI:** 10.3389/fneur.2017.00360

**Published:** 2017-07-24

**Authors:** Rani A. Sarkis, Kim C. Willment, Seth A. Gale, Barbara A. Dworetzky

**Affiliations:** ^1^Department of Neurology, Brigham and Women’s Hospital, Harvard Medical School, Boston, MA, United States

**Keywords:** elderly, epilepsy, Alzheimer’s, seizure semiology, aura

## Abstract

Seizures are a common co-morbidity during the course of Alzheimer’s disease (AD) and in a subset of patients may be one of the presenting symptoms. In this case series, we highlight three patients with recurrent medically refractory epileptic auras whose work up ultimately lead to the diagnosis of AD. All three patients underwent prolonged EEG, serial neuropsychological testing, FDG-PET, cerebrospinal fluid (CSF) AD biomarkers, and MRI. CSF biomarkers were particularly helpful in two cases. These cases highlight the importance of having a high index of suspicion for AD in new onset “idiopathic” epilepsy in the elderly.

## Introduction

Alzheimer’s disease (AD) is a progressive neurodegenerative condition characterized at its onset by cognitive difficulties usually in the domain of memory and/or executive dysfunction (1). Seizures are prevalent in around 28% of cases with AD and were long thought mostly to be a late manifestation of the disease process with the progression of neurodegeneration (2). However, more recent studies have revealed that in some cases, seizures related to AD may precede or coincide with the onset of the cognitive changes (3–5). Neuroimaging studies in patients with mild cognitive impairment (MCI) have highlighted hippocampal hyperactivation on fMRI (6), and treatment with the anti-seizure medication levetiracetam targeting this hyperactivation was found to improve memory task performance (7). At the network level, it seems that the early preclinical stages of AD are characterized by network hyperactivity with interneuron dysfunction central to the pathogenesis of the disorder (8).

Here, we present three patients (Table 1) identified from the outpatient epilepsy clinic at Brigham and Women’s Hospital who initially presented with medically refractory epileptic auras whose clinical course and subsequent evaluations were consistent with AD. The patients provided written consent for this publication.

**Table 1:** Clinical characteristics of the cases.

	Patient 1	Patient 2	Patient 3
Age at aura onset	64	70	70
Age at cognitive symptoms	64	73	70
Age at AD diagnosis	69	77	72
Aura semiology	Autonomic, abdominal, affective	Autonomic	Abdominal, autonomic
Aura frequency prior to meds	Several a week	Daily	Weekly
Aura frequency after meds	Once a week	Monthly	Monthly
Epilepsy risk factors	Encephalitis as a child	None	None
Family history	+AD in mother, sister	None	+AD in mother
Interictal/ictal EEG (age)	R T slowing (66) R hem szs (68), R T SW, L T LRDA	L T slowing (75) L T periodic discharges (76)	L T slowing and rare SW (age 71)
MRI findings	Bilateral hippocampal hyperintensities	Normal	Normal
FDG-PET	L > R T, R P-O hypometabolism	Normal	Normal
CSF			
WBC, protein, glucose	0, 29, 64	2, 44, 55	1, 49, 54
ATI, p-Tau	0.30, 68.95	1.50, 65.9	0.71, 102.45
Last anti-seizure regimen	CLZ, GBP, LTG	ZNS, PGB	LTG
Last CDR	1	0.5	0.5

*AD, Alzheimer’s disease; ATI, Aβ (1–42) tau index; CDR, clinical dementia rating; CLZ, clonazepam; CSF, cerebrospinal fluid; GBP, gabantin; Hem, hemispheric; L, left; LRDA, lateralized rhythmic delta activity; LTG, lamotrigine; P–O, parietal–occipital; PGB, pregabalin; R, right; SW, sharp wave; Szs, seizures; T, temporal; WBC, white blood cells; ZNS, zonisamide*.

### Case Series

#### Patient 1

**Figure 1:**
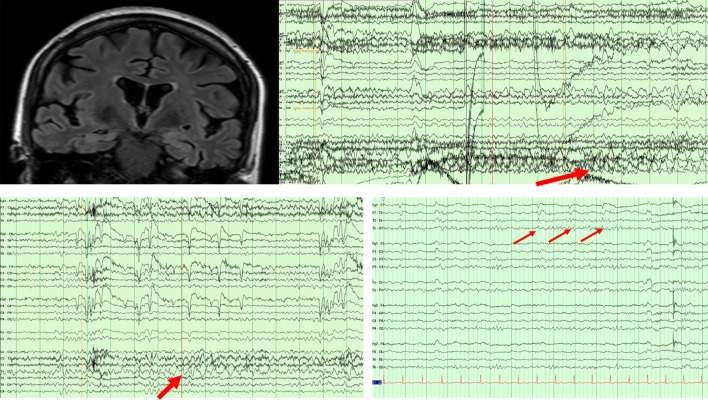
MRI FLAIR sequence revealing bitemporal FLAIR hyperintensities in patient 1. EEG (above) showing a right temporal seizure in patient 1. EEG (below left) showing left temporal rhythmic delta activity in patient 1. EEG (below right) showing periodic left temporal sharp waves in patient 2.

A 66-year-old woman presented with 2 years of progressive memory loss, changes in taste preference, and stereotyped episodes consisting of a “wave” of nausea and sweating, sometimes preceded by a visual hallucination. Over time, events were described as sudden, severe anxiety or feeling “repulsed” by seeing innocuous objects. Bilateral hippocampal hyperintensities were found on brain MRI and basic cerebrospinal fluid (CSF) assays, including an autoimmune panel (NMDA, VGKC, GAD65, GABA-B, AMPA, ANNA-1,2,3, AGNA-1, amphyphysin) were unremarkable. Ambulatory EEG revealed bitemporal, independent irregular slowing with no changes to the EEG background during her typical events of nausea and visual hallucinations. Episodes improved in frequency and duration with the addition of lamotrigine and gabapentin. An epilepsy monitoring unit (EMU) admission 4 years after her initial symptoms captured nine right hemispheric seizures (Figure 1), and left temporal lateralized rhythmic delta activity after medications were weaned. Clinical seizures decreased to 1 per week with dosage adjustments. An updated brain MRI was unchanged while an FDG-PET showed left >right temporal hypometabolism and mild posterior parietal–occipital hypometabolism (Figure 1). Repeat CSF revealed an elevated p-Tau level (68.95 pg/ml) and an Aβ (1–42) tau index (ATI) of 0.30 suggesting AD (9). At age 70, she continues to have weekly, affective auras despite three anti-seizure medications.

#### Serial Neuropsychological Testing

The patient was seen for neuropsychological evaluations at ages 66 and 68. The results of the first neuropsychological exam revealed high average estimated premorbid intellectual abilities [American version of the National Adult Reading Test Full-Scale Intelligence Quotient (FSIQ) = 115 (84th%ile)] and mild executive functioning difficulties, including trouble with planning and organization, problem solving flexibility, and response inhibition. She also had difficulty on a visual memory measure. The repeat neuropsychological exam (Table 2) revealed progressive decline primarily in the realm of executive functioning. She had greater difficulty with response inhibition, problem-solving flexibility, and divided attention/set-shifting. With regard to memory, she demonstrated a significant decline in word list learning delayed retrieval. Memory storage remained intact across memory measures. Confrontation naming was strong at both time points. Both phonemic and semantic verbal fluency on the Controlled Oral Word Reading Test were also impressive; however, she demonstrated a stable asymmetry with stronger performance on phonemic vs semantic trials. Moderate anxiety and mild depression were endorsed on self-report inventories at both time points. The primary pattern of progressive decline in executive dysfunction and verbal memory retrieval, in addition to the subtle evidence for semantic and visuoconstructional changes is a common neurocognitive profile for highly intelligent individuals presenting with early signs of a neurodegenerative disorder specifically AD (10).

**Table 2:** Serial neuropsychological testing.

	Pt 1	Pt 2	Pt 3
Premorbid FSIQ estimate	115 (84th%ile)	93 (32nd%ile)	108 (77th%ile)
	T1 | T2	T1 | T2	T1 | T2
WAIS-IV digit span total	24 (37th) | 23 (37th)	28 (75th) | 26 (63rd)	30(84th) | 22 (37th)
Trail Making Test B	82” (61st) | 116” (19th)	148 (34th) | 84 (84th)	176 (<1st) | 150 (<1st)
COWAT (FAS)	80 (>99th) | 69 (99th)	18 (7th) | 26 (21st)	45 (58th) | 43 (53rd)
Boston Naming Test	59 (84th) | 58 (75th)	40 (5th) | 43 (13th)	58 (75th) | 59 (79th)
ROCF copy	22 (<1st) | 20.5 (<1st)		16 (1st) | 21 (1st)
WAIS-IV Matrix Reasoning		16 (90th) | 10 (37th)	
**7-24 Spatial Memory Test**			
Immediate recall		24 (3rd) | 21 (1st)	
Delayed recall		5 (37th) | 2 (2nd)	
**Word list learning**	HVLT-R	HVLT-R	CERAD
Total recall (3 encoding trials)	26 (47th) | 21 (27th)	20 (27th) | 16 (8th)	17 (9th) | 16 (7th)
Delayed recall	10 (63rd) | 0 (<1st)	0 (<1st) | 4 (5th)	3 (1st) | 2 (<1st)
**ROCF**			
Immediate recall	11 (21st) | 7 (4th)		8.5 (14th) | 14 (53rd)
Delayed recall	10.5 (16th) | 7 (3rd)		8 (10th) | 10 (21st)

*CERAD, Consortium to Establish a Registry for AD; COWAT, Controlled Oral Word Association Test; FSIQ, Full-Scale Intelligence Quotient; HVLT-R, Hopkins Verbal Learning Test-Revised; ROCF, Rey–Osterrieth complex figure; WAIS, Wechsler Adult Intelligence Scale*.

#### Patient 2

A 75-year-old man presented with 5 years of near daily stereotyped episodes characterized by brief facial flushing (lasting seconds). He had been tried on several anti-seizure medications which he was unable to tolerate. He reported short-term memory difficulties for the past 2 years. He was later admitted to the EMU at age 76, and two auras were captured without epileptiform correlate although left temporal lateralized periodic discharges emerged during sleep (Figure 1). With the initiation of zonisamide and pregabalin, his aura frequency improved to monthly. Brain MRI and FDG-PET were unrevealing but CSF demonstrated elevated levels of p-Tau (65.9 pg/ml) and ATI of 1.50 one of which, the p-tau, suggests AD. Serum and CSF paraneoplastic panels were negative. A repeat brain MRI revealed cortical atrophy especially in the frontal and parietal regions. The patient’s seizure frequency at last follow-up remained stable, and he continued to complain of progressive cognitive decline, but remained independent in activities of daily living.

#### Serial Neuropsychological Testing

The patient was seen for neuropsychological evaluations at the ages of 75 and 76 (Table 2). His first exam revealed average estimated premorbid intellectual abilities [Wechsler Test for Adult Reading (WTAR) FSIQ = 93 (32nd%ile)] and a lateralizing profile to suggest greater involvement of the presumed language dominant left hemisphere. He demonstrated impairments in verbal fluency, confrontation naming, and word list learning delayed memory retrieval. The one exception to this lateralizing profile was impairment in spatial memory encoding. Other areas assessed included basic attention, orientation, aspects of executive function, and visuospatial skills were within expected range. His follow-up exam revealed persistent difficulties in word list learning delayed retrieval and non-verbal abstract reasoning and stable semantic processing/verbal retrieval weaknesses and spatial memory encoding impairments. Confrontation naming was impaired at both time points. Minimal symptoms of depression and anxiety were endorsed on self-report inventories also at both time points. Thus, there was evidence for progressive dysfunction within temporal–parietal systems (bilaterally).

#### Patient 3

A 71-year-old man presented with 1 year of stereotyped episodes, described as a rising abdominal sensation and facial flushing occurring twice weekly. He also began noticing short-term memory difficulties, which led him to retire from work. Brain MRI was normal. Ambulatory EEG over 72 h did not capture his auras but showed frequent, interictal left temporal irregular delta and rare left temporal sharp waves noted during sleep. Gabapentin over 4 months provided no benefit and he was transitioned to lamotrigine. An FDG-PET was normal. Serum and CSF paraneoplastic panels were negative, but a p-Tau of 102.45 and an ATI of 0.71 suggested AD. At last follow-up at age 72, his aura frequency had improved to one aura a month on a total lamotrigine dose of 150 mg.

#### Serial Neuropsychological Testing

The patient was seen for neuropsychological evaluations at the ages of 71 and 72 (Table 2). The first exam revealed average to high average estimated premorbid intellectual abilities [WTAR FSIQ = 108 (77th%ile)] and impairments in aspects of executive function, verbal memory retrieval, and semantic activation retrieval. His follow-up exam revealed significant declines in attention, visuospatial abilities, aspects of executive functioning, and memory encoding and retrieval. Visuoconstructional skills were impaired across tasks and appeared to extend beyond planning and organizational difficulties. Confrontation naming was strong, but he demonstrated continued difficulty with isolated aspects of semantic processing, specifically semantic activation retrieval on language tasks. Memory encoding and retrieval (both verbal and visual) were impaired, but there was limited evidence for storage loss. Minimal to mild symptoms of depression were endorsed on self-report inventories at both time points. These findings suggested progressive dysfunction in frontal, parietal, and temporal networks.

## Discussion

Studies have shown that seizures related to AD may precede or coincide with the onset of the cognitive decline (3–5). Our three cases suggest that elderly onset temporal epileptic auras may be a presenting symptom of AD. Two of our patients had a CSF profile typical of AD, while one patient did not have low amyloid levels in CSF, but had a pattern of atrophy on MRI and a neuropsychological profile suggestive of AD. At the University of California San Francisco series of 47 patients with seizures and AD, 7 had jamais vu/déjà vu, psychic phenomena, or sensory phenomena (4), while another case series from France (3) highlighted 13 cases of what they termed “epileptic prodromal AD,” 6 of whom had an “ascending aura” the majority with impaired awareness. The authors concluded that there is an epileptic variant of AD usually starting around the age of 60 with seizures as the initial symptom followed by cognitive complaints. A diagnosis of AD was often made at the MCI stage. Our case series confirms such a presentation and highlights the serial neuropsychological profile with detailed testing.

It is essential when evaluating patients with new onset “idiopathic” epilepsy in the elderly to maintain a high index of suspicion for AD. Autoimmune/pareneoplastic limbic encephalitis can have a similar presentation with high-frequency epileptic auras and seizures, but usually with a more acute symptomatology, inflammatory CSF, and/or MRI findings (11). Prolonged EEG can be quite helpful as epileptiform abnormalities may only show up in sleep. In fact, seizures may not be detected on scalp EEG if limited to deep cortical regions such as the hippocampus with only invasive testing revealing the ictal signature (12). FDG-PET in two of our cases was not particularly helpful, likely because it was done earlier in the disease process, where FDG-PET sensitivity for AD is often lower (13). We found CSF AD markers particularly helpful in two of our cases, and serial neuropsychological testing helpful in all three. While each of our cases had probable AD, the absence of neuropathogical tissue means that none of our patients fulfilled criteria for “definite” AD (14).

The pathophysiology underlying these three cases may indeed be deposition of beta-amyloid and tau leading to network hypersynchrony and temporal epileptic auras (15). Animal models of AD have documented epileptiform discharges and subclinical seizures; in a study of human amyloid precursor protein transgenic mice monitored with continuous EEG and hippocampal depth electrodes frequent focal or generalized discharges and non-convulsive seizures were seen (16). In another transgenic mouse model, serial EEG monitoring for 3 weeks revealed that up to 65% of the mice developed seizures during their lifetime (17). The genetic suppression of APP overexpression resulted in a normalization of EEG activity (18). Drug interventions in these models have found levetiracetam to be superior to sodium channel blockers in the suppression of the excitability and lead to an improvement of cognitive and behavioral performance (19). Similarly, decreasing tau in animal models of epilepsy has also been shown to attenuate neuronal hyperexcitability (20).

The presence of seizures and epileptiform abnormalities in humans confirms such a hypersynchrony and has been found to be highly prevalent in patients with AD monitored using EEG or MEG (21).

## Concluding Remarks

Recurrent medically refractory epileptic auras may be the presenting symptom of AD. With new onset “idiopathic” epilepsy in the elderly, it is important to maintain clinical suspicion for neurodegenerative disease, rather than focusing on seizure treatment alone.

## Ethics Statement

This study was carried out in accordance with the recommendations of Brigham and Women’s IRB.

## Author Contributions

RS: manuscript conception, manuscript drafting, data analysis, and final approval. KW, SG, and BD: manuscript revision, data analysis, and final approval.

## Conflict of Interest Statement

The authors declare that the research was conducted in the absence of any commercial or financial relationships that could be construed as a potential conflict of interest.
